# Glycemic indicators and mental health symptoms: results from the greater Beirut area cardiovascular cohort

**DOI:** 10.3389/fendo.2024.1347092

**Published:** 2024-10-16

**Authors:** Zahraa Mohammad Chamseddine, Mona P. Nasrallah, Hani Tamim, Lara Nasreddine, Martine Elbejjani

**Affiliations:** ^1^ Department of Epidemiology and Population Health, Faculty of Health Sciences, American University of Beirut, Beirut, Lebanon; ^2^ Department of Internal Medicine, American University of Beirut Medical Center, Beirut, Lebanon; ^3^ Clinical Research Institute, Department of Internal Medicine, Faculty of Medicine, American University of Beirut, Beirut, Lebanon; ^4^ College of Medicine, Al Faisal University, Riyadh, Saudi Arabia; ^5^ Department of Nutrition and Food Sciences, Faculty of Agricultural and Food Sciences, American University of Beirut, Beirut, Lebanon

**Keywords:** fasting blood glucose (FBG), HbA1c - hemoglobin A1C, depressive symptoms, anxiety symptoms, mental health, community-based sample, glycemic indicators, diabetes

## Abstract

**Introduction:**

Depression and anxiety present high and complex comorbidity with diabetes. One proposed explanation is that glycemic dysregulations and diabetes-related processes can influence mental health risk. We examined the associations of concurrent and prior glycemic indicators (Hemoglobin A1c (HbA1c) and fasting blood glucose (FBG) levels) with depression and anxiety symptoms in a community-based sample of middle-aged Lebanese adults.

**Methods:**

Data come from the Greater Beirut Area Cardiovascular Cohort (GBACC), with baseline and 5-year assessments of sociodemographic, lifestyle, and biological factors (n=198). Depression (Patient Health Questionnaire-9) and anxiety (General Anxiety Disorder-7) symptoms were assessed at follow-up. We investigated associations between glycemic indicators and continuous mental health scores using first linear and then piecewise regression models.

**Results:**

Adjusted piecewise regression models showed different associations with mental outcomes across glycemic indicators in the diabetic/clinical compared to the non-diabetic range: Among participants with <126 mg/dl baseline FBG, higher FBG levels in this range were significantly associated with lower depressive (beta=-0.12, 95%CI= [-0.207, -0.032]) and anxiety symptoms (beta=-0.099, 95%CI= [-0.186, -0.012]). In contrast, among participants with baseline FBG levels ≥126 mg/dl, higher FBG levels were significantly associated with higher anxiety symptoms (beta=0.055; 95%CI= 0.008, 0.102). Higher baseline FBG levels in the ≥126 mg/dl range showed a not statistically significant trend for higher depressive symptoms. Although not significant, baseline HbA1c levels showed similar patterns with negative associations with mental health symptoms in the <6.5% range.

**Discussion:**

Results show that FBG levels were associated with poorer mental health symptoms only in the clinical/diabetic range, and not in the normal range. Associations were observed with baseline glycemic indicators, highlighting potentially early and prolonged associations with mental health. Findings highlight the importance of clinical changes in glycemic indicators for mental health and motivate further research into the transition toward adverse associations between diabetes and mental health.

## 1 Introduction

Mental health disorders are a growing public health priority, affecting up to one billion people globally (1). Depression was the second and anxiety eighth leading cause of healthy life years lost to disability according to the Global Burden of Disease (GBD) in 2019 (2). This burden was largely exacerbated during the COVID-19 pandemic, with a 2020 Lancet review, reporting prevalence increase of 27.6% and 25.6% for major depressive and anxiety disorders respectively (3).

These worldwide prevalence and burden trends call for an improved understanding of the development of mental health disorders and for better recognition of specific higher-risk subgroups and trajectories. While the exact mechanisms underlying depression and anxiety remain unclear, accumulated data indicate that the risk of depression and anxiety is multifaceted and includes genetic, socioeconomic, environmental, lifestyle, and biological factors (4). Importantly, mental health disorders show high patterns of co-morbidity with other chronic disorders, underlining potential common biological and physiological processes. One consistently reported co-morbidity is that between depression and type II diabetes mellitus (T2DM), a common and serious chronic disease, ranked as the eighth leading cause of death and disability worldwide (2). According to the International Diabetes Federation (IDF), 425 million people had diabetes in 2017 and this number is anticipated to climb to 629 million by 2045 (5).

Both epidemiological and clinical studies show a higher prevalence of depression among people with diabetes; estimates from meta-analyses indicate that depression is twice more common in diabetic compared to non-diabetic populations (6–8). Moreover, data suggest that the course of depression among people with diabetes is more severe and complicated, due to underdiagnosis, under-treatment, and higher relapse occurrences (7, 9). Although less studied, data also suggest a link between diabetes and anxiety disorders, whereby patients with diabetes were reported to be 1.5 times more likely to develop severe anxiety than persons without diabetes (10–12), This suggests that diabetes-mental health links go beyond a specific disorder, further warranting a better understanding of how diabetes and its processes may impact mental health.

In parallel, several studies indicate that people with depression have an increased risk of T2DM (13). Combined, current evidence suggests a complex bi-directional relationship between depression and diabetes (14, 15), which is predominantly explained by three hypotheses: that shared common risk factors increase risk of both disorders simultaneously; that diabetes is a risk factor for depression; and that depression and stressful experiences may lead to diabetes (13, 16). One approach to better delineate this complex comorbidity is to investigate the more direct links between mental health symptoms and the biological building blocks of diabetes (10). However, studies investigating the links between depression and glycemic indicators have yielded mixed findings and have important limitations. First, most prior research consists of cross-sectional studies and do not include repeated assessments of glycemic indicators to investigate their timing and change in relation to mental health symptoms. Second, existing longitudinal studies have predominantly focused on patients with diabetes, where extreme changes in diabetes indicators may have already occurred, limiting the investigations of the relation of mental health symptoms across the non-diabetic to diabetic ranges. For instance, in a recent meta-analysis summarizing longitudinal studies, a bidirectional relationship has been reported between depressive symptoms and Hemoglobin A1c (HbA1c), with higher baseline HbA1c levels being associated with increased risk of probable depression; and higher baseline depressive symptoms associated with subsequent higher levels of HbA1c (17). Similar evidence is observed for anxiety symptoms, with studies reporting positive correlations between HbA1c, fasting blood glucose (FBG) levels and anxiety symptoms (18, 19). While these studies provide evidence for a link between depression and biological diabetes indicators, they are conducted in type 1, type 2, and mixed diabetic populations, and there is increasing interest and need for investigating how these relationships occur in the normal-to-pathological range of glycemic indicators (i.e., across the non-diabetic to diabetic range and in both people with and without diabetes) (17, 20–22). Reports of a positive association between pre-diabetic FBG/HbA1c levels and depressive symptoms (22) further highlight the need for investigating earlier associations with mental health illnesses across the spectrum of FBG/HbA1c variations -i.e., before the shift to more extreme and clinical ranges have occurred - to better assess relationships of glycemic deregulations and diabetes-related processes with depression and anxiety. For that, investigations in middle-aged adults can provide an added advantage to explore a time window where diabetes risk is changing (20).

This study aimed to examine the relationship of previous, concurrent, as well as changes in glycemic indicators (FBG and HbA1c) over five years with depression and anxiety symptoms in a community-based sample of middle-aged Lebanese adults.

## 2 Materials and methods

### 2.1 Study design and sample

This study was based on data from the Greater Beirut Area Cardiovascular Cohort study. Details of the study are described elsewhere (23, 24). Briefly, at baseline (2014), a sample of 501 adults were enrolled in the study following multistage probability sampling across the Greater Beirut area. Participants aged 18 and above and living in the Greater Beirut area were eligible to participate; pregnant women, dialysis patients, subjects with intellectual inability to understand the study and to provide informed consent were excluded (25, 26). Baseline data collection consisted of face-to-face interviews, anthropometric measurements, and extensive data on blood markers of cardiac and metabolic disorders. At the five-year follow-up (2019), participants were invited to participate in the second study wave. All participants who had consented at baseline to be re-contacted and who provided contact information (n=486) were called to participate in the follow-up, except for 8 subject who were no longer eligible to participate in the study; 198 completed the study follow-up examination. Of the 478 who were eligible to partake in the follow-up study, 36.1% were not successfully reached because of wrong phone numbers and 17.9% did not answer; 17.5% were too busy, 15.7% were not interested, 8.9% were too ill, and 3.9% had moved/traveled. A cohort flowchart is included in **Supplementary Figure S1**. Both study waves were approved by the Institutional Review Board at the American University of Beirut and all participants provided written consent at both study examinations.

### 2.2 Data collection

At baseline and follow-up, data on sociodemographic, socioeconomic, and lifestyle factors and medical history were collected through face-to-face interviews with trained data collectors; anthropometric and blood samples were also collected, through similar protocols at baseline and follow-up.

#### 2.2.1 Depression and anxiety symptoms

Data on mental health were only collected at the follow-up wave. Depressive symptoms were measured using the Patient Health Questionnaire-9 (PHQ-9) (27). The scale includes 9 self-reported items that assess the presence and severity of major depressive disorders based on the Diagnostic and Statistical Manual of Mental Disorders (DSM-IV) criteria in the past two weeks; each item is ranked in frequency on a 4-point Likert scale, with the total scores ranging from 0 to 27 (28) and higher score indicating higher depressive symptoms. The scale has been shown to have high internal consistency (Cronbach’s alpha between .86 and .88) (29) and high test reliability (Cronbach’s alpha between .84 and .95) (28, 30).

Anxiety symptoms were assessed using the Generalized Anxiety Disorder -7 (GAD-7) scale, a reliable and valid instrument widely used for assessing presence and severity of anxiety symptoms (31). The GAD-7 includes seven items, based on DSM-IV criteria (28), with each item’s frequency of occurrence over the past two weeks ranked on a 4-point Likert scale. Total scores range from 0 to 21, with higher scores indicating higher anxiety symptoms. The GAD-7 exhibits excellent internal consistency (Cronbach’s alpha between .89 and .92) (30–32).

Both scales were adapted and validated in Lebanon and Arab speaking communities (28) and the validated versions were used for data collection. We analyzed depressive and anxiety symptoms continuously (total scores of each the PHQ-9 and GAD-7 scales) as well as categorically, comparing those with elevated symptoms and those without using validated cut-offs [scores of 10 and more for each scale (12)].

#### 2.2.2 Glycemic indicators

The two main exposure variables of interest were fasting blood glucose (FBG) and glycosylated hemoglobin A1C (HbA1c), and we were interested in the baseline and follow-up values of these indicators, as well as their baseline to 5-year change measured as


(Value at baseline)−(Value at the 5–year follow‐up).

Glycemic indicators were based on blood draws collected at both the baseline and follow-up examination. Blood draws were split into two ethylenediaminetetraacetic acid tubes, one frozen for future studies and the other refrigerated and saved for HbA1c measurement within one week. The remaining blood was centrifuged with serum split into several 1 mL Eppendorf tubes, the one dedicated for FBG analysis was refrigerated and sent to be assayed immediately (on same day). HbA1c assessments were performed using the HPLC (Bio-Rad) and FBG assessments using the Enzymatic method (Cobas 6000, Roche). The same glycemic definitions were used at baseline at follow-up, according to the American Diabetes Association (ADA) guidelines: Diabetic FBG levels were considered as those equal and above 126 mg/dl; diabetic HbA1c levels were considered at 6.5% or higher. Presence of probable diabetes was defined as the presence of self-reported diabetes, taking diabetes medications, and/or either FBG≥126 mg/dl or HbA1c≥6.5% (48mmol/mol). Information on family history of diabetes was self-reported and categorized into presence of family history of diabetes (if any one of participant’s mother, father, or siblings had diabetes) versus no family history of diabetes.

#### 2.2.3 Covariates

The following covariates were selected to be accounted for in the analysis, based on an extensive literature review of the factors that are consistently associated with anxiety, depression and diabetes and that can be on common causal pathways for these conditions. Sociodemographic covariates included age (continuous variable), sex (men/women), and educational attainment (higher educational level including secondary/technical/university degree versus lower including no education/primary or intermediate school). Health characteristics included BMI (kg/m^2^), current smoking status (yes/no) (33), and physical activity (low, moderate and high) according to the International Physical Activity Questionnaire (IPAQ) scoring cut-offs (34). Participants self-reported whether they have been ever told by a doctor or healthcare professional that they have high blood pressure (hypertension: yes/no) at both study visits. We also considered the following self-reported medical conditions that are potentially relevant for both mental health disorders and diabetes: presence (yes/no) of dyslipidemia, coronary heart disease (CHD), cancer, thyroid, and stroke; a composite score was generated summarizing the number of metabolic conditions and chronic diseases (ranging from 0 to 4, with higher values indicating more conditions/comorbidities), to facilitate analysis as the prevalence of some disorders was low in the sample. The baseline (2014) values of the covariates were used for analyses involving baseline assessments of FBG and HbA1c, and follow-up (2019) values were used in analyses of follow-up FBG and HbA1c levels.

### 2.3 Statistical data analysis

Sample characteristics, mental health symptoms, and glycemic indicators (HbA1c and FBG) were described using mean and standard deviations for continuous variables and frequency distributions for categorical variables. We first investigated the correlation between the main exposures (HbA1c and FBG) at both study waves using Pearson correlations; similarly, we investigated the overlap in elevated depression and anxiety symptoms (using chi-square tests) and the correlation between continuous depression and anxiety scores using Pearson correlations.

For the main relationships of interest (glycemic indicators and mental health), we first investigated bivariate relationships between each of the baseline and follow-up FBG and HbA1c levels as well as the 5-year change in these levels with each of the depression and anxiety symptoms at follow-up (both continually and categorically (i.e., elevated symptoms yes/no), using linear and logistic regression analyses, respectively). We also investigated whether probable diabetes status at baseline and follow-up were related with depression and anxiety symptoms.

We investigated graphically the association of baseline and follow-up FBG and HbA1c levels with depressive and anxiety symptoms, using Locally Weighted Scatterplot Smoothing (Lowess) plots, which identified ranges across each of the HbA1c and FBG levels that showed differential relationships with mental health symptoms. As detailed in the results, relationships of FBG and HbA1c with mental health symptoms were different in the non-diabetic and diabetic ranges (below and above HbA1c of 6.5; below and above FBG of 126). Accordingly, we used segmented linear piecewise regression, a type of analysis that is efficient for scenarios of different patterns of associations specific ranges of the predictor variables; this method thus allowed to partition the glycemic indicators’ levels into two intervals (the non-diabetic and diabetic/clinical ranges), based on the ranges identified in the Lowess plots. We conducted adjusted piecewise regression models sequentially, first adjusting for age, sex, educational levels, BMI, smoking status, and hypertension; then, we further adjusted for number of medical conditions (dyslipidemia and number of chronic diseases), family history of diabetes, and physical activity. Analyses were performed for each glycemic indicator (FBG and HbA1c) and for each of the baseline and follow-up values of these predictors, aiming to assessing both cross-sectional association with mental health symptoms (follow-up data) and relationship of prior levels of these indicators (baseline) with mental health symptoms 5 years later. We used the covariates values that correspond to the year of the primary predictor in the model (e.g., baseline covariate values for baseline FBG levels). Regression coefficients (betas) and 95% confidence intervals were reported. The threshold for statistical significance was 5%. For each outcome, same analysis was repeated for the binary outcome classification (elevated symptoms yes/no) using logistic regression models (**Supplementary Table S1**) and results were concordant. Data analysis were performed using STATA 13.

## 3 Results

### 3.1 Sample characteristics

At five-year follow-up, mean age was 51.5 years (SD=13.38); 64.14% of the sample were women and 36.22% had higher educational attainment (**Table 1**). Average BMI was 30.43 kg/m^2^ (± 5.90) compared to 30.00 (± 5.85) kg/m^2^ at baseline. Current smoking and low physical activity were prevalent at both baseline and follow-up. Regarding medical history, 59.09% of participants had no chronic diseases at baseline while 45.45% reported no chronic diseases at follow-up; the most frequent diseases and conditions were dyslipidemia and hypertension and their prevalence increased at follow-up; presence of cancer and stroke history was stable over the 5 years with low prevalence of 2.53% and 1% respectively at both years (**Table 1**).

**Table 1:** Characteristics at baseline and follow-up of the Greater Beirut Area Cardiovascular Cohort sample (n=198).

		Baseline		5-years follow-up	
		n (%) or Mean ± SD	Missing n	n (%) or Mean± SD	Missing n
**Age**		46.96 ± 3.31	0	51.56 ± 13.38	0
**Sex**	Female	127 (64.14)	0	–	0
**Educational level**	Higher	59 (29.95)	0	71 (36.22)	2
Glycemic and diabetes indicators					
**FBG (mg/dl)**		109.08 ± 29.52	0	117.62 ± 39.76	3
**HbA1c (%)**		5.90 ± 1.15	0	5.95 ± 1.31	3
**Diabetes^¥^**	Yes	43 (21.27)	0	60 (30.30)	0
**Family history of diabetes**	Yes	107 (54.04)	0		
Lifestyle and health characteristics					
**BMI (kg/m^2^)**		30.00 ± 5.85	0	30.43 ± 5.90	1
**Current smoking**		78 (39.39)	0	80 (40.40)	0
**Hypertension**		38 (19.19)	0	72 (35.35)	0
**Dyslipidemia**		51 (25.76)	0	81 (40.91)	0
**Coronary heart disease**		17 (8.59)	0	30 (15.15)	0
**Stroke**		2 (1.01)	0	2 (1.01)	0
**Cancer**		5 (2.53)	0	5 (2.53)	0
**Thyroid disease**		24 (12.12)	0	27 (13.64)	0
**Number of medical conditions^€^**	None	117 (59.09)	0	90 (45.45)	0
	One	66 (33.33)	0	74 (37.37)	0
	Two	13 (6.57)	0	31 (15.66)	0
	Three	1 (0.51)	0	3 (1.52)	0
	Four	1 (0.51)	0	0	0
**Physical activity levels**	Low	92 (46.46)	0	104 (52.79)	1
	Moderate	73 (36.87)		70 (35.53)	
	High	33 (16.67)		23 (11.68)	
Mental health outcomes					
**Elevated depressive symptoms**	Yes	–	–	64 (32.3%)	0
**Total depression scores**		–	–	7.16 **±** 5.68	0
**Elevated anxiety symptoms**	Yes	–	–	53 (26.8%)	0
**Total anxiety scores**		–	–	6.57 **±** 5.55	0

^¥^Presence of diabetes is defined as FBG ≥ 126 or HbA1c ≥ 6.5 and/or self-reported diabetes and/or taking diabetic medication.

^€^Medical conditions include the presence of any of: Dyslipidemia, coronary heart disease, stroke, cancer, thyroid disease.

FBG, Fasting Blood Glucose; HbA1c, Hemoglobin A1c; BMI, Body Mass Index.

At baseline, 21.27% of the sample had diabetes and the prevalence increased to 30.30% at the five-year follow-up. This was accompanied with increases in FBG and HbA1c levels: Average FBG and HbA1c levels at follow-up were 117.62 mg/dl (± 39.76) and 5.95% (± 1.31%), compared to 109.08 mg/dl (± 29.52) and 5.9% (± 1.15%) at baseline, respectively.

With regards to mental health symptoms, 32.3% and 26.8% of participants had elevated depression and anxiety symptoms respectively. Average depression symptoms score was 7.16 (±5.68) and average anxiety symptoms score was 6.57 (± 5.55). Depression and anxiety scores were highly correlated (*ρ* = 0.69); 20.71% of the total sample had co-morbid elevated depression and anxiety symptoms. Most participants with elevated depression symptoms had co-morbid elevated anxiety symptoms (64.06%), and the majority of participants with elevated anxiety symptoms had co-morbid elevated depression symptoms (77.36%).

Bivariate associations between covariates of interest and total depression and anxiety scores (PHQ-9 and GAD-7 scores) are presented in **Supplementary Table S2**: Women had significantly higher depression and anxiety scores; higher education level was associated with significantly higher depression scores and with close-to-statistical significance higher anxiety scores. Baseline hypertension was associated with higher depressive symptoms (p=0.06); having a higher number of medical conditions at baseline and follow-up was associated with significantly higher depressive symptoms; anxiety scores were also higher with each additional condition at follow-up (p value=0.076).

### 3.2 Correlations among glycemic indicators

HbA1c and FBG levels showed positive, strong, and significant correlation at both study waves: at baseline (ρ = 0.81) and five-year follow-up (ρ = 0.84). Baseline HbA1c levels were strongly correlated with follow-up HbA1c levels (ρ = 0.79); baseline FBG levels were highly correlated with five-year follow-up FBG levels (ρ = 0.69) (**Table 2**).

**Table 2:** Correlation of fasting blood glucose (FBG) levels and Hemoglobin A1c (HbA1c) levels at baseline and five-year follow-up in the Greater Beirut Area Cardiovascular Cohort sample (n=198).

Glycemic indicators	Study wave	FBG Baseline	FBG 5-year follow-up	HbA1c Baseline	HbA1c 5-year follow-up
**FBG**	Baseline	1			
**FBG**	5-year follow-up	0.69**	1		
**HbA1c**	Baseline	0.81**	0.67**	1	
**HbA1c**	5-year follow-up	0.71**	0.84**	0.79**	1

**p-value <0.05.

### 3.3 Glycemic indicators and depressive and anxiety symptoms

Unadjusted linear regression analyses (**Table 3**) showed that baseline glycemic indicators were related to both mental health outcomes: higher baseline FBG levels were associated with higher depressive and anxiety symptoms (beta=0.024, 95% CI= [-0.002, 0.051], p-value=0.072 and beta=0.035, 95% CI= [0.009, 0.060], p-value=0.009, respectively); higher baseline HbA1c levels also showed a trend of positive association with depressive symptoms but without reaching statistical significance (beta=0.532, 95% CI = [-0.158, 1.223], p-value=0.130). Baseline diabetes status was associated with higher depression scores (beta=2.110, 95% CI= [0.195, 4.026], p-value=0.031) and with a trend for higher anxiety symptoms (beta=1.647, 95% CI = [-0.223, 3.52], p-value=0.08).

**Table 3:** Unadjusted linear regression models of baseline and five-year follow-up glycemic indicators and mental health symptoms.

		Depression symptoms (PHQ-9 scores)			Anxiety symptoms (GAD-7 scores)		
		beta	95% CI	P-value	beta	95% CI	P-value
**FBG**	Baseline	0.024	[-0.002 0.051]	0.072	0.035**	[0.009 0.060]	0.009
	5-year follow-up	0.009	[-0.009 0.029]	0.329	0.007	[-0.012 0.027]	0.473
**HbA1c**	Baseline	0.532	[-0.158 1.223]	0.130	0.460	[-0.214 1.135]	0.180
	5-year follow-up	0.126	[-0.490 0.742]	0.687	0.135	[-0.467 0.738]	0.661
**Change in FBG^¶^**		-0.008	[-0.036 0.018]	0.535	-0.024	[-0.051 0.003]	0.084
**Change in HbA1c^¶^**		-0.702	[-1.690 0.286]	0.163	-0.538	[-1.507 0.430]	0.274
**Diabetes, yes**	Baseline	2.110**	[0.195 4.026]	0.031	1.647	[-0.223 3.520]	0.085
	5-year follow-up	0.509	[-1.228 2.247]	0.564	-0.556	[-2.251 1.139]	0.519

^¶^Value at 5-year follow-up – Value at baseline

**p-value <0.05.

FBG, Fasting Blood Glucose; HbA1c, Hemoglobin A1c; PHQ-9, Patient Health Questionnaire 9; GAD-7, Generalized Anxiety Disorder 7.

5-year follow-up FBG and HbA1c levels were not associated with concurrent depressive symptoms (p-value>0.33) or anxiety symptoms (p-values>0.47). Change in HbA1c and FBG levels was also not significantly related to mental health symptoms.

**Figure 1:**
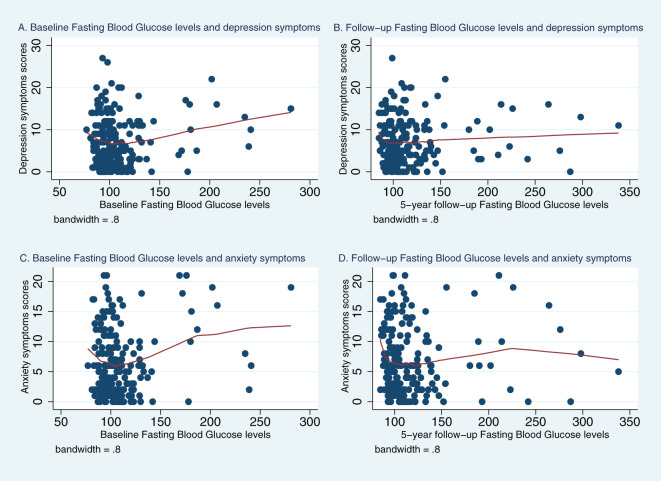
Lowess plots of the relationship of depression and anxiety symptoms with baseline and 5-year follow-up fasting blood glucose (FBG) levels. **(A)** Baseline Fasting Blood Glucose level and depression symptoms. **(B)** Follow−up Fasting Blood Glucose levels and depression symptoms. **(C)** Baseline Fasting Blood Glucose levels and anxiety symptoms. **(D)** Follow−up Fasting Blood Glucose levels and anxiety symptoms.

**Figure 2:**
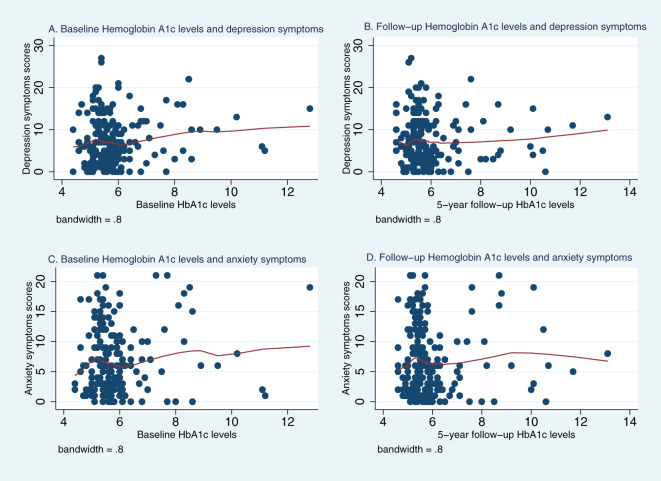
Lowess plots of the relationship of depression and anxiety symptoms with baseline and 5-year follow-up Hemoglobin A1c (HbA1c) levels. **(A)** Baseline Hemoglobin A1c levels and depression symptoms. **(B)** Follow−up Hemoglobin A1c levels and depression symptoms. **(C)** Baseline Hemoglobin A1c levels and anxiety symptoms. **(D)** Follow−up Hemoglobin A1c levels and anxiety symptoms.

**Figures 1**, **2** represent Lowess plots of the association of baseline and follow-up FBG (**Figure 1**) and HbA1c (**Figure 2**) levels with depression and anxiety symptoms. The FBG plots (**Figure 1**) suggest a change in the association, wherein a positive association is observed in the range of FBG>126 whereas below 126 associations have a negative trend, and this is observed for both baseline and follow-up FBG values and for both depression and anxiety symptoms. A similar trend is observed for HBA1c values, wherein above 6.5% HbA1c values, a positive linear trend between HbA1c and mental health symptoms is more apparent, whereas below 6.5%, the association either follows a plateau or no clear patterns of association.

Given that these trends were observed for both glycemic indicators and for both outcomes, we conducted segmented piecewise regressions separating the ranges of <126 and ≥126 for FBG and <6.5 and ≥6.5 for HbA1c (**Table 4**). Significant associations were observed between baseline FBG levels and mental health symptoms: In the <126 (non-diabetic) FBG range, higher baseline FBG levels were associated with lower depressive (beta=-0.093, 95%CI= [-0.0177, -0.009], p=0.03), and anxiety symptoms (beta=-0.095, 95%CI=[-0.1763, -0.0147]; p=0.02). In the diabetic ≥ 126 FBG range, higher baseline FBG levels were significantly associated with higher anxiety symptoms (beta=0.054; 95%CI= [0.007, 0.101]; p=0.026). Higher baseline FBG levels in the ≥126 range showed a non-significant trend for higher depressive symptoms (**Table 4**).

**Table 4:** Piecewise regression analysis of baseline and 5-year follow-up fasting blood glucose (FBG) and Hemoglobin A1c (HbA1c) levels and depression and anxiety symptoms.

Model	Glycemic Indicators		Depression symptoms (PHQ-9 scores)				Anxiety symptoms (GAD-7 scores)			
		Glycemic indicator range	beta	95% CI		p-value	beta	95% CI		p-value
Unadjusted Model 1	**FBG Baseline**	< 126	-0.093**	[-.0177	-0.009]	0.030	-0.095**	[-.1763	-.0147]	0.021
		≥126	0.038	[-0.011	0.087]	0.126	0.054**	[0.007	0.101]	0.026
	**FBG 5-year Follow-up**	< 126	-0.017	[-0.101	0.066]	0.684	-0.048	[-0.131	0.035]	0.255
		≥126	0.013	[-0.019	0.045]	0.436	0.018	[-0.014	0.050]	0.268
	**HbA1c Baseline**	< 6.5	-1.436	[-3.477	0.604]	0.167	-1.526	[-3.520	0.467]	0.133
		≥6.5	0.429	[-0.911	1.770]	0.528	0.488	[-0.821	1.798]	0.463
	**HbA1c 5-year Follow-up**	< 6.5	-0.989	[-3.204	1.226]	0.380	-1.617	[-3.773	0.538]	0.141
		≥6.5	0.525	[-0.688	1.738]	0.395	0.587	[-0.593	1.769]	0.328
Adjusted Model 2 ^¶^	**FBG Baseline**	< 126	-0.111**	[-0.199	-0.023]	0.013	-0.092**	[-0.179	-.0005]	0.038
		≥126	0.039	[-0.008	0.087]	0.103	0.055**	[0.008	0.102]	0.022
	**FBG 5-year Follow-up**	< 126	-0.025	[-0.113	0.063]	0.569	-0.041	[-0.130	0.046]	0.354
		≥126	0.014	[-0.017	0.045]	0.378	0.017	[-0.014	0.049]	0.274
	**HbA1c Baseline**	< 6.5	-2.062*	[-4.243	0.119]	0.064	-1.456	[-3.650	0.737]	0.192
		≥6.5	0.447	[-0.837	1.732]	0.493	0.519	[-0.773	1.811]	0.429
	**HbA1c 5-year Follow-up**	< 6.5	-2.089*	[-4.430	0.251]	0.080	-1.974*	[-4.296	0.348]	0.095
		≥6.5	0.469	[-0.703	1.642]	0.431	0.486	[-0.677	1.649]	0.411
Adjusted Model 3 ^¥^	**FBG Baseline**	< 126	-0.120**	[-0.207	-0.032]	0.008	-0.099**	[-0.186	-0.012]	0.026
		≥126	0.041*	[-0.006	0.088]	0.087	0.055**	[0.008	0.102]	0.021
	**FBG 5-year Follow-up**	< 126	-0.020	[-.1086	0.067]	0.647	-0.031	[-0.121	0.058]	0.494
		≥126	0.009	[-.0218	0.041]	0.543	0.013	[-0.018	0.045]	0.409
	**HbA1c Baseline**	< 6.5	-1.91*	[-4.120	0.293]	0.089	-1.269	[-3.496	0.956]	0.262
		≥6.5	0.517	[-0.769	1.804]	0.429	0.536	[-0.762	1.834]	0.416
	**HbA1c 5-year Follow-up**	< 6.5	-1.940	[-4.311	0.430]	0.108	-1.833	[-4.258	0.590]	0.137
		≥6.5	0.297	[-0.860	1.456]	0.613	0.302	[-0.882	1.486]	0.615

*p-value <0.10.

** p-value <0.05.

^¶^adjusted for age, sex, educational attainment, smoking, body mass index, and hypertension.

^¥^adjusted for age, sex, educational attainment, smoking, body mass index, hypertension, family history of diabetes, count of chronic diseases, and physical activity.

Similar conclusions were observed in models adjusted for age and sex and following further adjustment for educational attainment, BMI, current smoking, hypertension, family history of diabetes, number of medical conditions, and physical activity (**Table 4**). FBG levels at follow-up were not associated with concurrent mental health symptoms.

With regards to HbA1c, there was a similar trend for baseline HbA1c levels, with negative associations with mental health symptoms in the below 6.5 range and positive association in the ≥6.5 range; however, they did not reach statistical significance. Following adjustments for lifestyle and health indicators, the negative association between baseline HbA1c and depressive symptoms (beta= -1.91, 95%CI= [-4.21, 0.293]; p= 0.089) and between concurrent HBA1c levels and both depression (beta= -1.940, 95%CI= [-4.311,0.430]; p=0.108) and anxiety symptoms (beta= -1.833, 95%CI= [-4.528,0.590]; p= 0.137) in the below 6.5 range were more apparent (**Table 4**).

## 4 Discussion

### 4.1 Main findings

The current study aimed to assess the relationship of glycemic indicators with depression and anxiety scores in a community-based sample of middle-aged adults. In our sample, the prevalence of mental health problems was elevated with 32% of respondents having elevated depression and 26% having elevated anxiety symptoms; estimates from previous Ministry of Public Health reports (prevalence of 10-20% prior to the pandemic) also highlight the importance of and need for research on mental health in Lebanon (35). Our study revealed novel findings regarding the association of glycemic indicators and mental health. One important finding is that the association between FBG levels and mental health symptoms was differential across the non-diabetic to diabetic range of glycemic indicators, wherein only increases in FBG levels in the 126 and above range were associated with worsening depression and anxiety scores. Conversely increases in FBG levels, if they were in the below 126 range, were not associated with worst mental health scores and were related to lower mental health symptoms. A similar trend was observed for HbA1c levels, but it was close to statistical significance, with HbA1c levels in the below 6.5 range showing associations with lower mental health scores. This finding highlights a risk specific to reaching the diabetic range and worsening of glycemic indicators in the diabetic range for mental health outcomes. Another key finding was the association between baseline glycemic indicators with mental health symptoms, suggesting longer-term associations and indicating the value of early detection and management for diabetes and co-morbidities.

The finding of associations of FBG levels in the ≥126 range with poorer mental health outcomes are in line with other findings. According to a study conducted in New York that included 249 participants, a sample size that is close to ours, patients with diabetes showed a positive and significant correlation between FBG levels and PHQ-9 depression scores (36). Another study conducted in an Indian health care center among patients with diabetes also showed agreement with our result, where anxiety symptoms were positively associated with FBG (37). We note that very limited number of studies investigates FBG levels and most studies focused on HbA1c. Given the importance of both of these indicators in the context of diabetes, our study investigated both. We also note that associations of HbA1c with mental health outcomes followed the same trend of negative relationships with mental health scores in the non-clinical/diabetic HbA1c ranges (with close-to-statistical significance associations between baseline HbA1c <6.5 and lower mental health symptoms at baseline and follow-up (p ranging from 0.064 to 0.089 in models 2 and 3). In contrast, and unlike FBG levels, changes in HbA1c levels in the clinical/diabetic range were not associated with mental health symptoms. This could be explained in several ways. It is possible that the study’s sample size was limited in detecting some associations (despite the consistent pattern of associations for both HbA1c and FBG, at both times points, and with both outcomes), and that larger samples are needed to confirm the lack of association with mental health in the HbA1c clinical/diabetic range. It is possible that HbA1C, which reflects a 3-month average glucose, may lump fasting and post prandial glucose levels and capture different aspects than FBG levels. FBG levels in the non-diabetes state provide a more granular reflection of internal processes preceding the onset of diabetes, such as increased gluconeogenesis and insulin resistance, more so than the post prandial glucose which reflects beta cell function. It is also possible that increases in HbA1c levels within the diabetic range do not carry important consequences for mental health. Some studies found that HbA1c levels are associated with depression (15, 38–41) and anxiety (37) in people with diabetes whereas other studies found no association (42–44). A study among 514 participants in Iran did not find an association between poor glycemic control (high HbA1c values) and depression in people with diabetes (OR = 1.11, 95% CI = [0.87-1.57]) (45). While most of these studies are cross-sectional, other longitudinal studies also found no association. One longitudinal study over 5 years among 3762 patients with diabetes and another 3-year longitudinal study that aimed – as a secondary purpose – to investigate the relation between glycemic control and depression among adolescents showed that the relationship between depression and HbA1c was not significant after adjustment for confounders (17, 43, 46). Our results are in line with these findings, however the observed pattern of associations in our study emphasize that future studies will benefit from exploring the non-diabetic to diabetic ranges of HbA1c to better delineate particularities of this relationship.

In sum, our results show that increases in FBG and HbA1c levels were not linked to poorer mental health, as long as the glycemic values did not reach the clinical diabetes threshold. These findings can have important public health and clinical implications, as they suggest that it is not the gradual/cumulative increase in glycemic indicators’ values that is problematic in itself but rather it is entering the disease/pathological range. Further, these associations were observed directly with glycemic indicators (irrespective of diabetes status or treatment), suggesting that non-diabetic FBG and HbA1c levels (either naturally or controlled) may not impact mental health negatively. While our sample size limited thorough investigations of diabetes medication, we note that adjustment for diabetes medication did not change conclusions. Depression and anxiety have been linked to poorer glycemic control, adherence to treatment regimens, and dietary habits (38, 47, 48) further highlighting the need for future studies with larger samples and repeated assessments of both glycemic measures and mental health outcomes that can help assess their complex relationship and interplay through the role of adherence and success of treatment.

Another important finding of this study are the associations of the baseline diabetes-related measures with mental health scores, which were more apparent than associations with the 5-year follow-up diabetes-related measures, assessed at same time as the mental health scores. Changes in FBG and HbA1c levels were not related to mental health symptoms, which could be explained by the differential associations at different time points and across the diabetic and non-diabetic ranges. We also found that, similarly to FBG levels, diabetes status at baseline was associated with mental health outcomes. This could suggest a delayed or cumulative response between glycemic indicators and mental health outcomes, and that the relationship of higher glycemic levels and mental health may not appear instantly. This is in line with the nature of diabetes, a chronic disease involving complex interactions between multiple factors and consequences on several biological processes. Further, diabetes duration was associated with risk of depression in previous studies (48, 49). We note that patterns of associations were consistent at both baseline and follow-up and that HbA1c 5-year follow-up values in the non-diabetic range showed close to significance associations. We also note that values of both HBA1C and FBG were correlated across time, highlighting the stability of these indicators and raising the question of why some relationships will be observed at a specific time-point. Our analysis was limited by the one measurement of mental health and the absence of assessments at baseline, so we cannot exclude reverse causality and that higher previous glycemic indicators might have been observed among people with a prior mental health problem.

### 4.2 Strengths and limitations

To the best of our knowledge, this is the first study that address the relationship of mental health conditions across the normal to clinical range of glycemic indicators in a community sample. Prior studies were limited to specific samples with existing comorbidities (people with cardiovascular problems, diabetes 1 and 2, hypertensive, dialysis patients). In addition, this study is a cohort study with repeated assessments of glycemic indicators using rigorous and standardized data collection methods, whereas most previous studies relied on a one-time assessment of glycemic measures. Moreover, the time interval of 5 years between the two study waves allowed assessment of glycemic indicators and their relationships with mental health over a longer-term period. Our study was limited by the high drop-out rate, wherein 303 participants in the baseline wave were lost to follow-up. However, there was no major difference between the responders and non-responders (23). It is also important to note that the primary cause for loss to follow-up was due to the inability to contact because of the change in their contact information. This suggests that, despite the reduction in sample size, the follow-up sample was still representative of the baseline sample and that the drop-out did not cause major systematic differences and selection bias. The drop in sample size may have hindered the detection of smaller magnitude associations and the performance of some sub-group analysis (e.g., controlled diabetes versus uncontrolled diabetes). Furthermore, the study sample remains a selective sample recruited from the capital and its surroundings, and thus it is not representative of the general population in Lebanon. Another important limitation is that the study included only one follow-up wave and importantly one assessment of mental health at the follow-up visit. This hindered the assessment of temporal relationships between glycemic indicators and mental health symptoms. The one-time assessment of mental health also makes it difficult to rule out reverse causality and the scenario that baseline mental health may have impacted baseline and subsequent glycemic markers, as discussed above. At the same time, the lack of cross-sectional association of mental health with glycemic indicators and presence of diabetes at follow-up suggests that it is possible that mental health at baseline may be similarly unrelated to baseline glycemic indicators; and, that the associations of FBG and diabetes with mental health might be more delayed and prolonged in time. Longitudinal studies with several repeated assessments of both glycemic and mental health measures are needed to better describe their temporal and longitudinal associations and to identify shifts to the clinical range and earlier associations. Another limitation concerns potential measurement error in assessing mental health given their subjective nature. Moreover, most of the other chronic diseases were self-reported, and thus limited in capturing undiagnosed or unknown prior occurrences of disease and residual confounding. Finally, stress and traumatic exposures (whether previous such as war exposures or exposure to current hardships) were not assessed in the study and; these exposures are prevalent in the Lebanese context and may influence both mental health and the occurrence of chronic diseases.

## 5 Conclusion

In summary, results from this community-based sample showed that adverse associations with higher glycemic levels and poorer mental health were not observed in the normal range of these indicators, but rather only in the clinical/diabetic range of FBG. Moreover, this association was observed with prior and not concurrent glycemic levels strengthening the rationale for longitudinal investigations of the relationship between glycemic indicators and mental health symptoms that can help identify temporal and earlier associations and their timing with regards to clinical/diabetic changes in glycemic indicators. A better understanding of the complex co-morbidity between diabetes and mental health disorders can have significant implications for these highly prevalent and burdensome conditions, particularly for improving their prevention, management, and consequences. Advancing this knowledge can aid in developing two-dimension strategies for managing diabetes and mental health simultaneously, which can be particularly important in low-resourced settings such as Lebanon. Our work also puts forward important questions regarding the clinical course of glycemic indicators, as only the clinical range was associated with depression and anxiety symptoms, advocating for prioritizing medical and lifestyle interventions for diabetes and glycemic control to better improve the consequences and mental health co-morbidities for patients with diabetes.

## Data Availability

The raw data supporting the conclusions of this article will be made available by the authors, without undue reservation.
