# Supplementary material for: Glycemic indicators and mental health symptoms: results from the greater Beirut area cardiovascular cohort
Source: Front Endocrinol (Lausanne). 2024 Oct 16;15:1347092. doi: 10.3389/fendo.2024.1347092 (PMC11521852; doi:10.3389/fendo.2024.1347092)
Supplement: Supplementary file 1 [file DataSheet1.docx]

| Supplementary Table 1. Piecewise logistic regression analysis of baseline and 5-year follow-up fasting blood glucose and HbA1c levels and elevated depression and anxiety symptoms(n=198) | | | | | | | | | | |
| --- | --- | --- | --- | --- | --- | --- | --- | --- | --- | --- |
|  |  |  | **Elevated depression symptoms** | | | | **Elevated anxiety symptoms** | | | |
|  |  |  | **OR** | **95% CI** | | **p-value** | **OR** | **95% CI** | | **p-value** |
| Unadjusted Model | **FBG Baseline** | ***< 126*** | 0.970 | [0.937 | 1.004] | 0.092 | 0.956 | [0.919 | 0.994] | 0.024* |
|  |  | ***≥126*** | 1.012 | [0.992 | 1.032] | 0.222 | 1.014 | [0.995 | 1.034] | 0.142 |
|  | **FBG 5-year Follow-up** | ***< 126*** | 1.007 | [0.975 | 1.041] | 0.634 | .9837 | [0.949 | 1.018] | 0.359 |
|  |  | ***≥126*** | 1.006 | [0.994 | 1.018] | 0.271 | 1.004 | [0.992 | 1.016] | 0.469 |
|  | **HbA1c Baseline** | ***< 6.5*** | 0.645 | [0.309 | .3477] | 0.244 | 0.526 | [0.241 | 1.149] | 0.107 |
|  |  | ***≥6.5*** | 1.320 | [0.841 | 2.075] | 0.227 | 1.179 | [0.761 | 1.824] | 0.459 |
|  | **HbA1c 5-year Follow-up** | ***< 6.5*** | .8714 | [0.402 | 1.886] | 0.727 | .4220 | [0.178 | 0.999] | 0.050* |
|  |  | ***≥6.5*** | 1.246 | [0.856 | 1.815] | 0.251 | .9645 | [0.633 | 1.468] | 0.886 |
| Adjusted Model 2 ^¶^ | **FBG Baseline** | ***< 126*** | 0.959 | [0.921 | 0.998] | 0.043* | 0.950 | [0.947 | 1.025] | 0.024* |
|  |  | ***≥126*** | 1.013 | [0.990 | 1.036] | 0.253 | 1.016 | [0.992 | 1.018] | 0.148 |
|  | **FBG 5-year Follow-up** | ***< 126*** | 0.999 | [0.963 | 1.036] | 0.967 | 0.985 | [1.309 | 6.853] | 0.472 |
|  |  | ***≥126*** | 1.009 | [0.995 | 1.023] | 0.170 | 1.005 | [0.974 | 1.031] | 0.387 |
|  | **HbA1c Baseline** | ***< 6.5*** | 0.336 | [0.125 | 0.901] | 0.030* | 0.385 | [0.298 | 1.393] | 0.068 |
|  |  | ***≥6.5*** | 1.080 | [0.642 | 1.817] | 0.770 | 1.01 | [0.921 | 1.043] | 0.946 |
|  | **HbA1c 5-year Follow-up** | ***< 6.5*** | 0.659 | [0.243 | 1.788] | 0.413 | 0.314 | [0.358 | 1.517] | 0.041* |
|  |  | ***≥6.5*** | 1.375 | [0.831 | 2.273] | 0.215 | 0.994 | [0.910 | 4.283] | 0.982 |
| Adjusted Model 3 ^¥^ | **FBG Baseline** | ***< 126*** | 0.953 | [0.914 | 0.995] | 0.027* | 0.945 | [0.902 | 0.989] | 0.015* |
|  |  | ***≥126*** | 1.014 | [0.991 | 1.037] | 0.234 | 1.017 | [0.995 | 1.039] | 0.136 |
|  | **FBG 5-year Follow-up** | ***< 126*** | 1.000 | [0.963 | 1.039] | 0.989 | 0.99 | [0.951 | 1.029] | 0.608 |
|  |  | ***≥126*** | 1.008 | [0.994 | 1.022] | 0.284 | 1.003 | [0.989 | 1.016] | 0.694 |
|  | **HbA1c Baseline** | ***< 6.5*** | 0.346 | [0.124 | 0.965] | 0.089 | 0.384 | [0.133 | 1.110] | 0.077 |
|  |  | ***≥6.5*** | 1.092 | [0.645 | 1.851] | 0.429 | 1.006 | [0.617 | 1.642] | 0.981 |
|  | **HbA1c 5-year Follow-up** | ***< 6.5*** | 0.533 | [0.177 | 1.601] | 0.262 | 0.248 | [0.072 | 0.856] | 0.027* |
|  |  | ***≥6.5*** | 1.323 | [0.805 | 2.174] | 0.269 | 0.892 | [0.537 | 1.483] | 0.660 |
| *p-value <0.05  ^¶^ adjusted for age, sex, educational attainment, smoking, body mass index, and hypertension  ¥ adjusted for age, sex, educational attainment, smoking, body mass index, hypertension, family history of diabetes, count of chronic diseases, and physical activity | | | | | | | | | | |

***Supplementary Material***

| **Supplementary Table 2. Linear bivariate regression models of the main outcomes (depressive and anxiety symptoms) with socio-demographic, lifestyle and health characteristics at baseline and 5-year follow-up (n=198)** | | | | | | | | |
| --- | --- | --- | --- | --- | --- | --- | --- | --- |
|  |  | | **Depression (PHQ-9) Scores** | | | **Anxiety (GAD-7) Scores** | | |
|  |  | | **Beta** | **95% CI** | ***p*** | **Beta** | **95% CI** | ***p*** |
| **Socio-Demographic Characteristics** | | | | |  |  |  | |
| **Age** | | Baseline | 0.025 | [ -0.034 0.085] | 0.398 | -0.003 | [-0.060 0.057] | 0.954 |
|  |  | 5-year follow-up | 0.024 | [-0.035 0.084] | 0.422 | -0.002 | [-0.061 0.056] | 0.927 |
| **Sex, female** | |  | 3.589 | [ 2.001 5.177] | 0.000***** | 2.690 | [1.108 4.271] | 0.000***** |
| **Education, higher** | | Baseline | -2.150 | [-3.877 -0.423] | 0.015***** | -1.271 | [-2.973 0.430] | 0.142 |
|  |  | 5-year follow-up | -2.382 | [-4.025 -0.738] | 0.005* | -1.504 | [ -3.110 0.101] | 0.066**^#^** |
| **Covariates: Lifestyle and Health Characteristics** | | | | |  | | | |
| **Body Mass Index** | | Baseline | 0.095 | [-0.041 0.231] | 0.170 | 0.017 | [ -0.116 0.151] | 0.798 |
|  |  | 5-year follow-up | 0.090 | [-0.045 0.230] | 0.190 | 0.012 | [-0.120 0.145] | 0.853 |
| **Hypertension** | | Baseline | 1.916 | [-0.094 3.928] | 0.062**^#^** | 1.312 | [-0.659 3.284] | 0.191 |
|  |  | 5-year follow-up | 1.165 | [-0.489 2.818] | 0.166 | 0.980 | [-0.635 2.595] | 0.233 |
| **Number of medical conditions** | | Baseline | 1.614 | [0.474 2.755] | 0.006***** | 0.636 | [-0.495 1.767] | 0.269 |
|  |  | 5-year follow-up | 2.100 | [1.111 3.088] | 0.000***** | 0.903 | [-0.096 1.901] | 0.076**^#^** |
| **Currently Smoking** | | Baseline | 0.177 | [-1.458 1.813] | 0.831 | -0.920 | [-2.511 0.670] | 0.255 |
|  |  | 5-year follow-up | -0.397 | [-2.024 1.231] | 0.631 | -0.726 | [-2.313 0.859] | 0.367 |
| **Physical activity levels** | | |  |  |  |  |  |  |
| ***moderate*** | | Baseline | -0.813 | [ -2.574 0.948] | 0.364 | -0.386 | [ -2.089 1.317] | 0.665 |
| ***high*** | |  | 0.355 | [ -1.920 2.636] | 0.759 | 2.126 | [ -0.078 4.330] | 0.059**^#^** |
| ***moderate*** | | 5-year follow-up | -1.315 | [-3.003 0.372] | 0.126 | -1.341 | [ -3.013 0.329] | 0.115 |
| ***high*** | |  | -0.456 | [ -2.971 2.059] | 0.721 | 1.187 | [ -1.302 3.677] | 0.348 |

*p-value <0.05

^#^p-value <0.1

**Supplementary Figure 1. Greater Beirut Area Cardiovascular Cohort Flowchart**


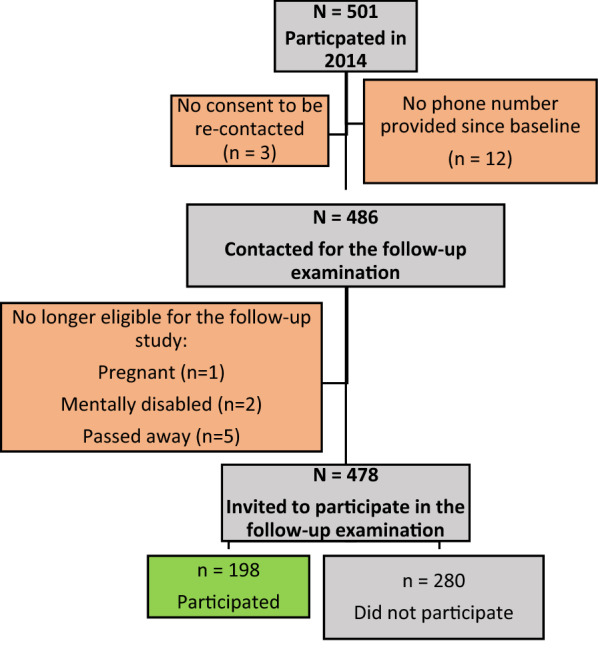


Nasrallah MP, Elbejjani M, Nasreddine L, Chami H, Ismaeel H, Fleifel M, Al Zahraa Chokor F, Tamim H. Incidence of diabetes and its predictors in the Greater Beirut Area: a five-year longitudinal study. Diabetol Metab Syndr. 2022 May 4;14(1):67. doi: 10.1186/s13098-022-00833-w. PMID: 35509100; PMCID: PMC9066987.
